# Validity and Reliability of the Japanese Version of the Newest Vital Sign: A Preliminary Study

**DOI:** 10.1371/journal.pone.0094582

**Published:** 2014-04-24

**Authors:** Takamichi Kogure, Masahiko Sumitani, Machi Suka, Hirono Ishikawa, Takeshi Odajima, Ataru Igarashi, Makiko Kusama, Masako Okamoto, Hiroki Sugimori, Kazuo Kawahara

**Affiliations:** 1 Department of Health Policy Science, Tokyo Medical and Dental University, Graduate School of Medical and Dental Science, Tokyo, Japan; 2 Department of Anesthesiology and Pain Relief Center, The University of Tokyo Hospital, Tokyo, Japan; 3 Department of Pain and Palliative Medicine, The University of Tokyo Hospital, Tokyo, Japan; 4 Department of Public Health and Environmental Medicine, The Jikei University School of Medicine, Tokyo, Japan; 5 Department of Health Communication, School of Public Health, The University of Tokyo, Tokyo, Japan; 6 Japanese Red Cross Kanto-Koshinetsu Block Blood Center, Tokyo, Japan; 7 Department of Drug Policy and Management, Graduate School of Pharmaceutical Sciences, The University of Tokyo, Tokyo, Japan; 8 Laboratory of Pharmaceutical Regulatory Sciences, Graduate School of Pharmaceutical Sciences, The University of Tokyo, Tokyo, Japan; 9 Department of Applied Biological Chemistry, Graduate School of Agricultural and Life Sciences, The University of Tokyo, Tokyo, Japan; 10 Department of Preventive Medicine, Graduate School of Sports and Health Sciences, Daito Bunka University, Saitama, Japan; Tokyo Metropolitan Institute of Medical Science, Japan

## Abstract

Health literacy (HL) refers to the ability to obtain, process, and understand basic health information and services, and is thus needed to make appropriate health decisions. The Newest Vital Sign (NVS) is comprised of 6 questions about an ice cream nutrition label and assesses HL numeracy skills. We developed a Japanese version of the NVS (NVS-J) and evaluated the validity and reliability of the NVS-J in patients with chronic pain. The translation of the original NVS into Japanese was achieved as per the published guidelines. An observational study was subsequently performed to evaluate the validity and reliability of the NVS-J in 43 Japanese patients suffering from chronic pain. Factor analysis with promax rotation, using the Kaiser criterion (eigenvalues ≥1.0), and a scree plot revealed that the main component of the NVS-J consists of three determinative factors, and each factor consists of two NVS-J items. The criterion-related validity of the total NVS-J score was significantly correlated with the total score of Ishikawa et al.'s self-rated HL Questionnaire, the clinical global assessment of comprehensive HL level, cognitive function, and the Brinkman index. In addition, Cronbach's coefficient for the total score of the NVS-J was adequate (alpha = 0.72). This study demonstrated that the NVS-J has good validity and reliability. Further, the NVS-J consists of three determinative factors: “basic numeracy ability,” “complex numeracy ability,” and “serious-minded ability.” These three HL abilities comprise a 3-step hierarchical structure. Adequate HL should be promoted in chronic pain patients to enable coping, improve functioning, and increase activities of daily living (ADLs) and quality of life (QOL).

## Introduction

Pain is defined as “an unpleasant sensory and emotional experience associated with actual or potential tissue damage, or described in terms of such damage” [1]. Pain is the most common patient-reported complaint in clinical practice, and is strongly associated with quality of life (QOL). Therefore, pain has been suggested as an important QOL indicator for patients with chronic illness (e.g., cancer) [2]. In clinical practice, patients' pain recognition and persistency are profoundly influenced by the strength of noxious stimuli and affective status, as well as various other factors [e.g., medical knowledge, social skills, activities of daily living (ADLs), economic status, interpersonal relationships]. The biopsychosocial model proposes that clinical pain management must incorporate psychological and social factors, along with biological variables [3]. In this model, pain is considered an interactive and psychophysiological pattern of behaviors that cannot be separated into distinct, independent psychosocial and physical components. Numerous studies support the usefulness of cognitive behavioral therapy and other psychological approaches to chronic pain management, in addition to those that support a pharmacotherapeutic approach [4], [5]. These psychological approaches yield similar outcomes, and commonly focus on educating patients with chronic pain to build coping skills and improve functioning. Successful treatment with pharmacotherapies requires the education of chronic pain patients regarding proper drug administration, side effects, and communicating with their physicians about unrelieved pain and prescription changes [6]. Thus, it is important to educate patients on the management of chronic pain. For example, opioids are prescribed to alleviate patients' chronic pain and improve their overall functioning. However, concerns regarding opioid abuse, addiction, adverse outcomes (e.g., respiratory depression and/or deep sedation from overdosing, and withdrawal symptoms from unintended discontinuation), and tolerance have been increasing. To address such concerns, chronic pain patients need to have adequate numeracy skills to ensure that they consume the right amounts of opioids. In other words, it is highly important that their physicians teach them how to count and take the correct number of pills dutifully.

Health literacy (HL) refers to the capacity to obtain, process, and understand basic health information and services, and is necessary to make appropriate health decisions. In other words, HL is a social skill that embodies the ability to access necessary information in order to maintain and promote better health [7]. More specifically, it refers to the ability to read, understand, and use health care information to make decisions and follow treatment instructions. From the viewpoint of health care professionals, patients need to possess a particularly sophisticated level of understanding to receive the care they need, and lower HL is commonly found among older adults and patients with chronic illnesses [8]. Lower HL has been associated with lengthier hospitalizations, greater use of emergency care, a lower rate of screening examinations, poorer medication compliance, and a lower ability to interpret labels and health messages, as well as lower overall health status and higher mortality among older adults [9]. Several HL assessment tools have already been developed. One such assessment is the Newest Vital Sign (NVS), which is comprised of 6 questions about an ice cream nutrition label and assesses HL numeracy skills [10]. HL numeracy skills facilitate adherence to medication regimens [11]. This is particularly important for opioid medications, where adequate adherence to dosing schedules is necessary to avoid unfavorable consequences (e.g., respiratory depression, addiction, and withdrawal symptoms upon abrupt discontinuation). Assessment of HL numeracy skills is consequently of great importance in clinical practices for treating chronic pain [12]. English, Turkish, Dutch, and Spanish versions of the NVS have already been validated in primary care patients; however, a highly necessary Japanese version has yet to be validated. In the present study, we developed and validated a Japanese version of the NVS (NVS-J) in patients with chronic pain. While the original NVS assessments were conducted via face-to-face interviews, the NVS-J was designed as a questionnaire available for routine use in a variety of situations.

## Materials and Methods

### Participants

This study was approved by the Institutional Ethics Committee, Faculty of Medicine, The University of Tokyo (#3678), and consistent with the Declaration of Helsinki. A unique aspect of the NVS is that it can potentially be used to screen for limited numeracy skills. The original NVS was validated in primary care patients. However, as we mentioned earlier, numeracy skills are vital to patients with chronic pain who are using opioid analgesics. Therefore, we focused on chronic pain patients in our research.

A subset of patients who had been seen more than three times in our outpatient clinic, the Department of Anesthesiology and Pain Relief Center, The University of Tokyo Hospital, were enrolled in the study. During the study period of January–February 2012, the participants eligible for recruitment were randomly selected from the appointment logs of the attending physicians. All of the participants reported pain of an intensity of 3 or higher out of 10 on an 11-point numerical rating scale (NRS: 0 = no pain, 10 = worst pain imaginable), and the attending physicians evaluated their pain as necessitating continuous treatment. Participants with cultural or language barriers, or poor mental health statuses, that prevented them from understanding or responding to the questionnaires were excluded from this study. Among 44 identified eligible patients, 43 provided oral informed consent to participate in the study, and completed the questionnaires. Demographic data were obtained on each participant through the self-report questionnaire [i.e., age, sex, height, body weight, occupation, intensity of pain (NRS), smoking history (Brinkman index = daily number of cigarettes * year), and education level].

### Measures

All patients were asked to complete the following 4 questionnaires: 1) the NVS-J; 2) a simple dementia screening test that assessed cognitive functioning (a total score of 12 or less out of 15 indicated possible dementia) [13]; 3) the Brief Pain Inventory (BPI) (Japanese version) for assessing ADLs [14]; and 4) a self-rated HL Questionnaire (HLQ) by Ishikawa et al., in which functional, communicative, and critical HL were assessed separately, with the total score of all three HL perspectives indicating an individual's comprehensive HL level [15]. Further, the attending physicians of each participant completed a clinical global impression scale of participants' comprehensive HL levels (CGI-HL) that consisted of a 5-point Likert-type scale (1 = “very poor,” 2 = “poor,” 3 = “fair,” 4 = “moderate,” 5 = “good”), on the basis of the following appraisals: 1) the participant always keeps his/her consultation appointments, 2) the participant understands the psycho-education concept about chronic pain management presented by the attending physician, 3) the participant can adequately adhere to medication regimens, 4) the participant can answer open questions, and 5) the participant can communicate coherently with the attending physician.

### Development of a Japanese version of the NVS

Translation and cross-cultural adaptation of the NVS-J was performed in accordance with the established guidelines [16], [17]. First, a forward translation of the original NVS into Japanese involving independent translations by a professional native Japanese translator and bilingual Japanese physician was obtained. Then, an expert committee including specialists in pain management, public health, and methodology, synthesized the two translations. Finally, two native English translators, who were uninformed about the nature of the study, completed back-translations of the translated NVS; thereafter, the back-translations were sent to an expert committee to detect cultural bias. When the NVS-J was deemed free of cultural bias, it was considered complete and suitable for administration to participants.

### Data analysis

A score of two or less on the CGI-HL was considered in this study as indicative of low HL. Sensitivity and specificity ratios, as well as the stratum-specific likelihood ratio (SSLR) were then calculated for the NVS-J score of each participant. The cut-off point for the NVS-J was set for screening purposes on the basis of these parameters and the area under the receiver operating characteristic (ROC) curve.

#### Feasibility

The feasibility of the NVS-J was determined by analyzing the number of unanswered questions.

#### Validity

Construct validity was established through an exploratory factor analysis with principal components extraction. The Kaiser criterion (eigenvalues >1.0) and scree plot were used to determine the number of factors. Criterion-related validity was assessed through the calculation of a Pearson correlation coefficient between the dementia screening score, BPI, NRS, NVS-J, HLQ, and physicians' impressions. The following are generally accepted rankings for coefficients: 1.0–0.81 (excellent), 0.80–0.61 (very good), 0.60–0.41 (good), 0.40–0.21 (fair), and 0.20–0 (poor) [18].

#### Reliability

Internal consistency was measured with Cronbach's alpha. Alpha coefficients of a magnitude ≥0.70 were considered evident of adequate scale reliability at the level of group comparisons [19]. Repeatability was assessed by a test-retest method. Intra-class correlation coefficients (ICCs) between test and retest scores were calculated based on data from participants who reported no symptom changes between the times of the two surveys. Coefficients >0.80 were considered indicative of excellent reliability [20].

All statistical analyses were performed using the Statistical Package for the Social Sciences (SPSS version 11.0) software.

## Results

### Participant characteristics

The sociodemographic and clinical characteristics of the participants are displayed in Table 1. The total score distribution of the NVS-J is presented in Table 2. The percentage of participants who answered correctly is shown for each NVS-J question in Table 3.

**Table 1:** Participant demographics.

	Mean	SD
**Age (yrs)**	64.5	14.4
**Male/Female**	25/18	
**Height**		
**Male (cm)**	167.2	6.5
**Female (cm)**	151.1	6.9
**Weight**		
**Male (kg)**	64.4	10.6
**Female (kg)**	47.8	8.4
**BMI**		
**Male**	23.0	3.4
**Femle**	20.9	3.3
**Brink Mann Index**	247.3	480.1

**Table 2:** Distribution of total NVS-J scores.

NVS-J score	0	1	2	3	4	5	6
**Number (n = 43)[%]**	13 [30.2]	7 [16.3]	4 [9.3]	8 [18.6]	4 [9.3]	6 [14.0]	1 [2.3]

**Table 3:** Percentage of correct answers for each NVS-J question.

	Kcal	Cup	Gram	%	Allergy	Reason
**Correct (%)**	37.2	18.6	51.2	25.6	41.9	37.2

### Validity

Factor analysis with promax rotation, using the Kaiser criterion (eigenvalues ≥1.0), and a scree plot revealed that the main component of the NVS-J consists of three determinative factors that constitute 100% of the variance (Table 4). The first of these determinative factors consisted of the first and second questions, and was termed “basic numeracy ability,” which referred to the capacity of participants to perform a simple calculation. The second factor consisted of the third and fourth questions, and was termed “complex numeracy ability,” which referred to the ability of participants to extract necessary information from a nutrition label and perform complex calculations. Finally, the third factor consisted of the fifth and sixth questions, and was termed “serious-minded ability,” which referred to the ability of participants to make reasonable health-related decisions. This factor was assessed by instructing participants to imagine they had been diagnosed with an allergic condition and asking whether they would consider avoiding allergenic foods.

**Table 4:** Factor analysis of the NVS-J.

Factor analysis	Factor1	Factor2	Factor3
**Kcal**	0.831	−0.179	−0.031
**Gram**	0.709	0.210	0.046
**%**	−0.182	0.828	−0.029
**Cup**	0.238	0.640	0.017
**Allergy**	0.000	−0.040	0.855
**Reason**	−0.012	0.043	0.834

In the analysis of criterion-related validity, the total NVS-J score was significantly correlated with the total HLQ score (p = 0.004, R = 0.43), functional HL score in the HLQ (p = 0.009, R = 0.39), and profoundly with the CGI (p<0.0001, R = 0.72). Furthermore, we observed that the NVS-J was significantly correlated with cognitive function (p = 0.016, R = 0.37) and the Brinkman index of smoking history (p<0.05, R = −0.30). These results also indicated the criterion-related validity of the NVS-J. Conversely, the total score of the NVS-J did not demonstrate any correlation with communicative and critical HL scores in the HLQ (p = 0.064, R = 0.39; p = 0.11, R = 0.25; respectively), body mass index (p = 0.79, R = −0.042), or pain intensity (p = 0.98, R = −0.004).

### Reliability

Cronbach's coefficient for the total NVS-J score was adequate (alpha = 0.72). We were able to recruit 18 participants for a test-retest study, all of whom reported no changes in their symptoms. The data for each participant were evaluated. The average period between the two surveys was 12.2 weeks [standard deviation (SD): 1.7]. A significant correlation between the two surveys was demonstrated by a Pearson's correlation analysis (p<0.001, R = 0.82), which suggests good reproducibility. The applicable rate is shown in Table 5. There were no unanswered questions on any surveys administered during either time point in the data that were analyzed.

**Table 5:** Applicable rate of the respective questions.

	Kcal	Cup	Gram	%	Allergy	Reason
**Applicable rate (%)**	90.1	86.0	79.1	74.4	81.4	79.1

### Cut-off point for low HL

The area under the ROC curve for the CGI-HL was 0.87. The theoretical maximum of this value is 1.00, which indicates perfect discrimination. A score of 2 on the NVS-J showed very high sensitivity (94.7%) but moderate specificity (75.0%); a score of 1 showed high sensitivity (84.2%) and relatively high specificity (83.3%). Further, the SSLR score of 1 was the maximum (5.05) among all the scores (Table 6). Therefore, a score of 1 on the NVS-J would be the suitable clinical cut-off point for screening purposes of low HL. This is compatible with the original cut-off point.

**Table 6:** Stratum-specific likelihood ratios for NVS-J cut-off scores by the CGI-HL.

Score of the NVS-J	Sensitivity	Specificity	Stratum-specific likelihood ratio (SSLR)
0	47.4	87.5	3.79
1	84.2	83.3	5.05
2	94.7	75.0	3.79
3	94.7	41.7	1.62
4	100	29.2	1.41
5	100	4.17	1.04
6	100	0.00	1.00

## Discussion

This study demonstrated that the NVS-J has good validity and reliability. The results obtained in this study were comparable to those in previous studies [10], [21]. With regard to criterion-related validity, significant correlations between the NVS-J, HLQ, and the CGI-HL by the physicians were observed. Furthermore, the NVS-J score was significantly correlated with cognitive function and smoking history, suggesting that the NVS-J would reflect overall numeracy ability and health practices. With regard to construct validity, we conducted a factor analysis and found that the six items of the NVS-J consist of three determinative factors, which can be defined as “basic numeracy ability,” “complex numeracy ability,” and “serious-minded ability.” A factor analysis of this nature has not yet been attempted with regard to the NVS. One was not performed in the original NVS study conducted by Barry et al. (2005) in the US, or in the validation study of the screen by Rowlands et al. (2013) in the UK [10], [21]. The factor analysis was fundamental in revealing covert psychometric properties of the NVS-J and the relationships between them. Here, we compared the present factor structure of the NVS-J to the HLQ. The HLQ assesses three components of HL: functional, communicative, and critical HL [15]. Functional HL refers to the ability to read and comprehend basic medical information. Communicative HL denotes the ability to extract important information and independently apply that information to personal health maintenance. Communicative HL is thus more advanced than functional HL, but still relatively basic. Critical HL refers to the extent to which individuals can thoroughly examine the necessity and suitability of medical information, and use that information to make decisions about personal health maintenance. These three HL abilities comprise a 3-step hierarchical structure. The present three extracted factors of the NVS-J are likely consistent with these core HL abilities in the HLQ [15].

In fact, our research revealed correlations between patients' NVS-J scores and total scores on the HLQ. NVS-J scores were also associated with functional HL, which is the fundamental subscale of the HLQ. These results indicate that the NVS-J has good criterion-related validity in evaluating overall HL and fundamental HL. On the other hand, NVS-J scores were not correlated with scores on the communicative and critical subscales of the HLQ. Its potential use for detecting limited numeracy skills makes the NVS one of a kind, as the HLQ cannot currently be used to ascertain such skills in individuals. Therefore, the NVS-J can be used independently or on its own to evaluate HL, especially numeracy skills.

Further, the distribution of scores attained by participants on the NVS-J, detailed in Table 2, varied from the one observed on the original NVS. However, our analysis utilizing ROC Curves clearly demonstrated that a score <2 on the NVS-J had moderately high sensitivity (84.2%) and specificity (83.3%) for predicting limited literacy, consistent with assessments by patients' attending physicians. This cut-off point was similar to that used in the original NVS study, in which the researchers also observed that scoring <4 could predict adequate literacy based on the stratum-specific likelihood ratios they obtained. However, our ratios (see Table 6) did not enable us to clearly categorize individuals in terms of whether they had adequate or robust health literacy. Therefore, differing from the original NVS, the present NVS-J could predict limited literacy when scores were <2 with a moderately high degree of specificity, but could not separate patients with adequate health literacy from those who had high health literacy.

Individuals with limited health literacy are less knowledgeable about their health problems [22]–[27], endure lengthier hospitalizations [28], [29], pay higher health care costs [30], [31], and are less healthy [32]–[36] than those with adequate or high health literacy. Health information can be tailored for delivery to patients in an understandable format, provided patients have adequate health literacy. Additionally, patients with low health literacy have poor knowledge of pain medications, including their proper use and intake. Opioid analgesics are potent and thus commonly prescribed for chronic pain treatment; however, these drugs carry significant dependence and abuse risk for a portion of patients. Health care professionals should consequently be trained to recognize patterns of opioid abuse and misuse, and educate chronic pain patients on proper opioid administration. Training and ongoing education should be provided for chronic pain patients on effective dosing schedules and risks of pain medications, particularly opioids, that have a high potential for misuse, abuse, dependence, and life-threatening withdrawal symptoms. Patients should be required to demonstrate adequate numeracy ability prior to receiving an opioid prescription intended for self-administration. Furthermore, chronic pain patients should be capable of understanding the specifics of non-pharmacological coping strategies that may improve functioning, ADLs, and QOL. Given that HL encompasses these basic cognitive abilities, HL assessment is essential for chronic pain patients, as well as patients suffering from other chronic illnesses (e.g., congestive heart failure, diabetes mellitus, and asthma).

### Limitations

The sample size of this study was small. In the original Newest Vital Sign (NVS) study by Barry et al. (2005) that enrolled 250 participants [10], the validity of the NVS was not directly associated with patients' health literacy (HL) levels as evaluated by health care professionals. Instead, by including the Test of Functional Health Literacy in Adults (TOFHLA), which can be used to assess medical linguistic problems [37], the original NVS demonstrated sensitivity and specificity for detecting low levels of HL. However, we felt that a conventional test/assessment utilizing medical words might be insufficient for detecting low HL levels in our study population (chronic pain patients). We thus decided to evaluate HL by also asking patients' attending physicians to complete a clinical global impression scale of patients' comprehensive HL levels, as they had expertise in educating patients on health and helping them understand health related information in their clinical practice. Because of this, the number of participants was fairly limited, but we were able to more precisely screen for limited HL with a higher degree of sensitivity and specificity. While the results of our preliminary study demonstrated the validity of the NVS-J in screening for limited HL, further research is required. From our pilot data, we could calculate the ideal sample size for more confirmatory investigations of its validity.
